# Supplementary material for: Physical Mechanism of Nonlinear Spectra in Triangene
Source: Molecules. 2023 Apr 26;28(9):3744. doi: 10.3390/molecules28093744 (PMC10180230; doi:10.3390/molecules28093744)
Supplement: Supplementary file 1 [file molecules-28-03744-s001.zip › molecules-2334765-Supplementary.pdf]

molecules

MDPI

Article

Physical Mechanism of Nonlinear Spectra in Triangene

Na Zhang [1,†], Weijian Feng [1,†], Hanbo Wen [1,†], Naixing Feng [2,3,4,5,*], Hao Sheng [1,*], Zhixiang Huang [2,3,4,5] and Jingang Wang [1]

1 Liaoning Provincial Key Laboratory of Novel Micro-Nano Functional Materials, College of Science, Liaoning Petrochemical University, Fushun 113001, China
2 The Key Laboratory of Intelligent Computing and Signal Processing, Ministry of Education, Anhui University, Hefei 230601, China
3 Anhui Province Key Laboratory of Target Recognition and Feature Extraction, Lu'an 230601, China
4 The Information Materials and Intelligent Sensing Laboratory of Anhui Province, Anhui University, Hefei 230601, China
5 The Key Laboratory of Electromagnetic Environmental Sensing of Anhui Higher Education Institutes, Anhui University, Hefei 230601, China
* Correspondence: fengnaixing@gmail.com (N.F.); shenghao@lnpu.edu.cn (H.S.)
† These authors contributed equally to this work.

Contents:

Figure S1. Single photon absorption spectra of triangulene spin chains with different structures.

Figure S2. Two-photon absorption spectra of TSCs with N=3,4-1,4-2,6c.

Figure S3. Electronic circular dichroism (a) of TSCs increasing periodically with size in one-dimensional direction, and electronic circular dichroism (b,c) of TSCs with two different shapes consisting of 4 and 8 triangulene units, respectively.

Figure S4. The spin density of the triangulene spin chains and the corresponding spin population of each segment.

Figure S5. Atomic coloring diagram of the contribution of atomic spin populations to a single electron in a triangulene spin chains, with red representing a positive contribution and blue representing a negative contribution.

Figure S6. N=2TSCs molecular model, atomic species and corresponding atomic number.

Table S1. Functional and basis groups and Cartesian coordinates for Gaussian optimization tasks with N=3TSCs.

Table S2. Functional and basis groups and Cartesian coordinates in Gaussian optimization tasks for N=4-1TSCs.

Table S3. Functional and basis groups and Cartesian coordinates in Gaussian optimization tasks for N=4-2TSCs.

Table S4. Functional and basis groups and Cartesian coordinates in Gaussian optimization tasks for N=6cTSCs.

Table S5. Atomic population of N=2TSCs

Citation: Zhang, N.; Feng, W.; Wen, H.; Feng, N.; Sheng, H.; Huang, Z.; Wang, J. Physical Mechanism of Nonlinear Spectra in Triangene. *Molecules* **2023**, *28*, 3744. https://doi.org/10.3390/molecules28093744

Academic Editors: Jinpeng Li, Jun Xu, Bin Wang and Wenhua Gao

Received: 25 March 2023
Revised: 21 April 2023
Accepted: 25 April 2023
Published: 28 April 2023

Copyright: © 2023 by the authors. Licensee MDPI, Basel, Switzerland. This article is an open access article distributed under the terms and conditions of the Creative Commons Attribution (CC BY) license (https://creativecommons.org/licenses/by/4.0/).

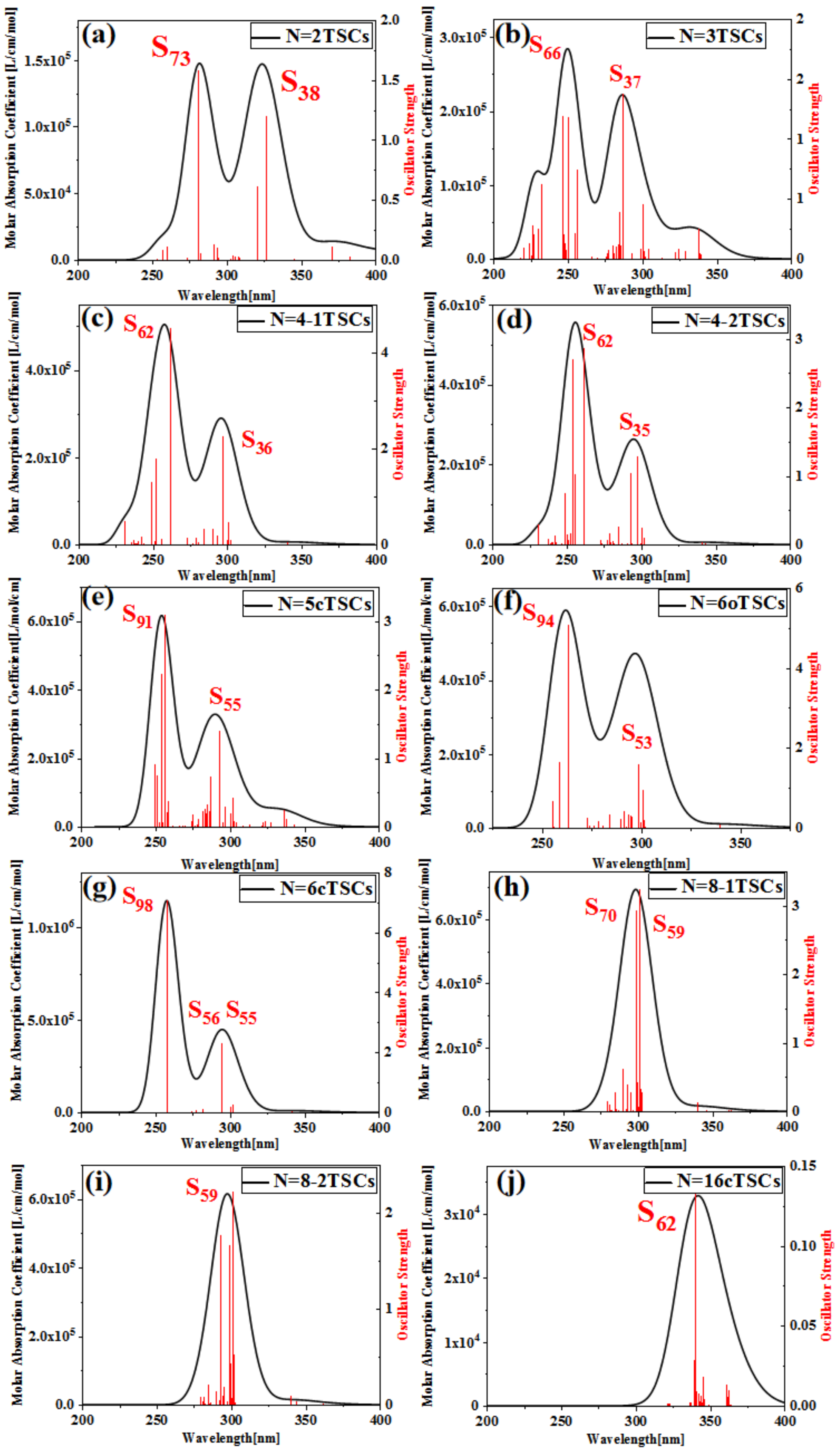

Figure S1. Single photon absorption spectra of triangulene spin chains with different structures.

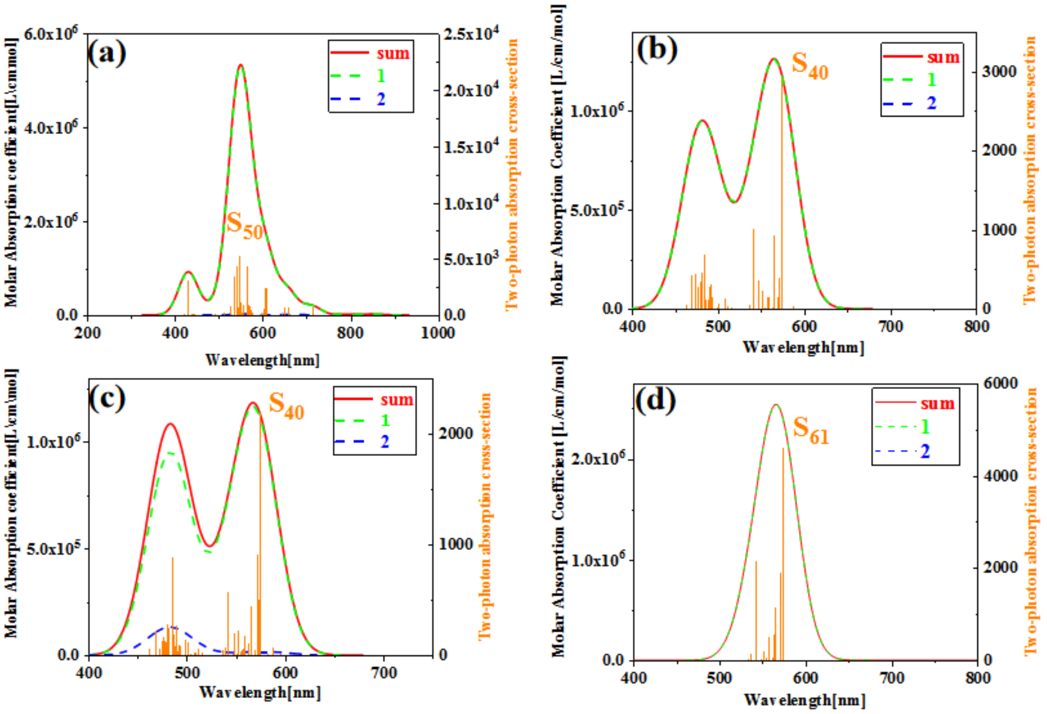

Figure S2. Two-photon absorption spectra of TSCs with N=3,4-1,4-2,6c.

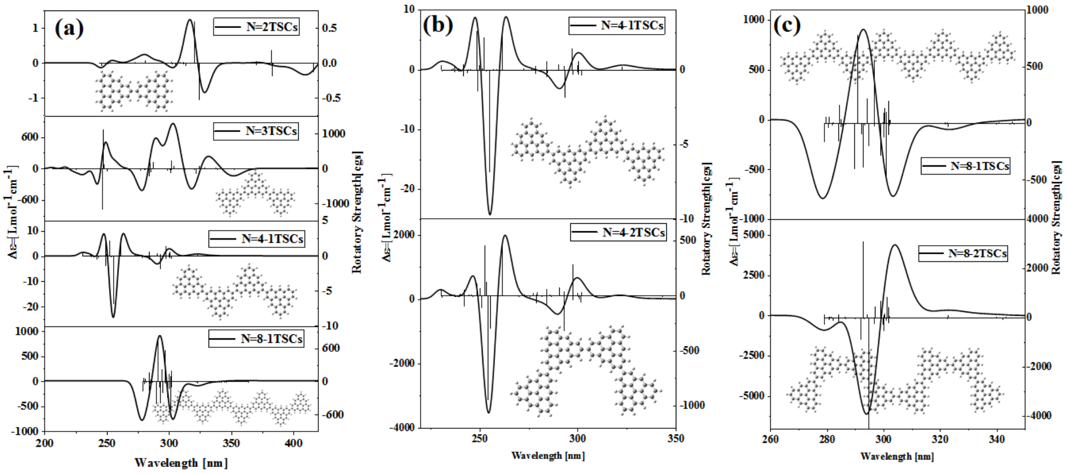

Figure S3. Electronic circular dichroism (a) of TSCs increasing periodically with size in one-dimensional direction, and electronic circular dichroism (b,c) of TSCs with two different shapes consisting of 4 and 8 triangulene units, respectively.

Table S1. Functional and basis groups and Cartesian coordinates for Gaussian optimization tasks with N=3TSCs.

#p opt pbe1pbe def2svp em=gd3bj			
0 1			
C	-4.7386	2.3421	0
C	-3.9686	1.0085	0
C	-2.4286	1.0085	0
C	-1.6586	2.3421	0
C	-2.4286	3.6758	0
C	-3.9686	3.6758	0

C	-0.1186	2.3421	0
C	0.6514	1.0085	0
C	-0.1186	-0.3252	0
C	-1.6586	-0.3252	0
C	0.6514	-1.6589	0
C	2.1914	-1.6589	0
C	2.9618	-0.3276	0.046
C	2.1914	1.0085	0
C	-2.4286	-1.6589	0
C	-3.9686	-1.6589	0
C	-4.7386	-0.3252	0
C	-0.1186	-2.9926	0
C	-1.6586	-2.9926	0
C	-4.7386	-2.9926	0
C	-3.9686	-4.3262	0
C	-2.4286	-4.3262	0
C	4.5016	-0.3262	0.0192
C	5.2703	1.0082	0.0058
C	6.81	1.0096	-0.0211
C	7.5811	-0.3234	-0.0345
C	6.8125	-1.6578	-0.0211
C	5.2727	-1.6592	0.0057
C	9.1209	-0.322	-0.0613
C	9.8895	1.0124	-0.0748
C	9.1184	2.3454	-0.0613
C	7.5787	2.344	-0.0345
C	7.5836	-2.9908	-0.0345
C	9.1233	-2.9894	-0.0614
C	9.892	-1.655	-0.0748
C	11.4317	-1.6536	-0.1016
C	12.2029	-2.9865	-0.115
C	11.4342	-4.3209	-0.1016
C	9.8944	-4.3223	-0.0748
C	11.4293	1.0138	-0.1016
C	12.2004	-0.3192	-0.115
C	9.8871	3.6797	-0.0747
C	11.4269	3.6811	-0.1016
C	12.198	2.3482	-0.115
H	-5.8786	2.3421	0
H	-1.8586	4.6631	0
H	-4.5386	4.6631	0
H	0.4514	3.3294	0

H	2.7614	-2.6456	-0.0341
H	2.7622	1.9947	-0.034
H	-5.8786	-0.3252	0
H	0.4514	-3.9798	0
H	-5.8786	-2.9926	0
H	-4.5386	-5.3135	0
H	-1.8586	-5.3135	0
H	4.6994	1.9949	0.0157
H	4.7037	-2.647	0.0157
H	7.0079	3.3307	-0.0245
H	7.0145	-3.9785	-0.0246
H	13.3427	-2.9855	-0.1349
H	12.005	-5.3077	-0.1116
H	9.3254	-5.3101	-0.0649
H	13.3402	-0.3181	-0.1349
H	9.3163	4.6665	-0.0648
H	13.3378	2.3492	-0.1349
C	12.3022	5.142	-0.0979
C	11.607	6.516	-0.0814
C	12.4485	7.8052	-0.116
C	13.9854	7.7204	-0.1669
C	14.6806	6.3464	-0.1833
C	13.839	5.0572	-0.1488
C	16.2174	6.2616	-0.2343
C	17.059	7.5508	-0.2688
C	16.3638	8.9249	-0.2524
C	14.827	9.0097	-0.2015
C	14.1317	10.3837	-0.185
C	12.5949	10.4685	-0.1341
C	11.7533	9.1793	-0.0996
C	17.2054	10.2141	-0.287
C	18.7422	10.1293	-0.3379
C	19.4374	8.761	-0.3551
C	18.5958	7.466	-0.3198
C	14.9733	11.6729	-0.2196
C	16.5101	11.5881	-0.2705
C	11.8997	11.8426	-0.1177
C	12.7413	13.1318	-0.1522
C	14.2781	13.047	-0.2032
H	10.4693	6.5788	-0.0437
H	14.3537	4.04	-0.161
H	16.7321	5.2444	-0.2465

H	10.6157	9.2421	-0.0618
H	19.3652	11.0837	-0.3629
H	20.5751	8.7001	-0.3939
H	19.1126	6.4499	-0.3315
H	17.1331	12.5425	-0.2961
H	10.762	11.9053	-0.0799
H	12.2266	14.1489	-0.14
H	14.9011	14.0014	-0.2287

Table S2. Functional and basis groups and Cartesian coordinates in Gaussian optimization tasks for N=4-1TSCs.

#p opt pbe1pbe def2svp em=gd3bj			
0 1			
C	-26.3801	-8.7157	0.0002
C	-27.5843	-8.021	0.0002
C	-27.5844	-6.631	0.0002
C	-26.3802	-5.936	0.0002
C	-25.176	-6.6309	0.0001
C	-25.1759	-8.0209	0.0001
C	-23.9721	-5.9363	0.0001
C	-23.9726	-4.5465	0
C	-25.1767	-3.8512	0.0001
C	-26.3803	-4.5461	0.0002
C	-27.5839	-3.8513	0.0002
C	-28.7879	-4.5467	0.0002
C	-28.7883	-5.9365	0.0002
C	-29.9917	-3.8518	0.0002
C	-29.9913	-2.4615	0.0001
C	-28.7875	-1.766	0
C	-27.5838	-2.4609	0.0001
C	-25.177	-2.4608	0
C	-23.9733	-1.7657	-0.0001
C	-22.7694	-2.4612	-0.0001
C	-22.7689	-3.8515	-0.0001
C	-26.3804	-1.7655	0
H	-26.38	-9.7157	0.0002
H	-28.4503	-8.5211	0.0003
H	-24.3099	-8.5209	0.0001
H	-23.1061	-6.4363	0
H	-29.6543	-6.4366	0.0003
H	-30.8577	-4.3517	0.0002
H	-30.8574	-1.9616	0
H	-28.7877	-0.766	0

H	-23.9732	-0.7657	-0.0001
H	-21.9028	-4.3513	-0.0001
H	-26.3804	-0.7655	0
C	-17.8804	4.5234	0.0002
C	-16.6762	3.8287	0.0002
C	-16.6761	2.4387	0.0002
C	-17.8803	1.7437	0.0002
C	-19.0845	2.4386	0.0001
C	-19.0846	3.8286	0.0001
C	-20.2884	1.744	0.0001
C	-20.2879	0.3542	0
C	-19.0838	-0.3411	0.0001
C	-17.8802	0.3538	0.0002
C	-16.6766	-0.341	0.0002
C	-15.4726	0.3544	0.0002
C	-15.4722	1.7442	0.0002
C	-14.2688	-0.3405	0.0002
C	-14.2692	-1.7308	0.0001
C	-15.473	-2.4263	0
C	-16.6767	-1.7314	0.0001
C	-19.0835	-1.7315	0
C	-20.2872	-2.4266	-0.0001
C	-21.4911	-1.7311	-0.0001
C	-21.4916	-0.3408	-0.0001
C	-17.8801	-2.4268	0
H	-17.8805	5.5234	0.0002
H	-15.8102	4.3288	0.0003
H	-19.9506	4.3286	0.0001
H	-21.1544	2.244	0
H	-14.6062	2.2443	0.0003
H	-13.4028	0.1594	0.0002
H	-15.4728	-3.4263	0
H	-20.2873	-3.4266	-0.0001
H	-22.3577	0.159	-0.0001
H	-17.8801	-3.4268	0
C	-9.2801	-8.7157	0.0002
C	-10.4843	-8.021	0.0002
C	-10.4844	-6.631	0.0002
C	-9.2802	-5.936	0.0002
C	-8.076	-6.6309	0.0001
C	-8.0759	-8.0209	0.0001
C	-6.8721	-5.9363	0.0001

C	-6.8726	-4.5465	0
C	-8.0767	-3.8512	0.0001
C	-9.2803	-4.5461	0.0002
C	-10.4839	-3.8513	0.0002
C	-11.6879	-4.5467	0.0002
C	-11.6883	-5.9365	0.0002
C	-12.8917	-3.8518	0.0002
C	-12.8913	-2.4615	0.0001
C	-11.6875	-1.766	0
C	-10.4838	-2.4609	0.0001
C	-8.077	-2.4608	0
C	-6.8733	-1.7657	-0.0001
C	-5.6694	-2.4612	-0.0001
C	-5.6689	-3.8515	-0.0001
C	-9.2804	-1.7655	0
H	-9.28	-9.7157	0.0002
H	-11.3503	-8.5211	0.0003
H	-7.2099	-8.5209	0.0001
H	-6.0061	-6.4363	0
H	-12.5543	-6.4366	0.0003
H	-13.7577	-4.3517	0.0002
H	-11.6877	-0.766	0
H	-6.8732	-0.7657	-0.0001
H	-4.8028	-4.3513	-0.0001
H	-9.2804	-0.7655	0
C	-0.7804	4.5234	0.0002
C	0.4238	3.8287	0.0002
C	0.4239	2.4387	0.0002
C	-0.7803	1.7437	0.0002
C	-1.9845	2.4386	0.0001
C	-1.9846	3.8286	0.0001
C	-3.1884	1.744	0.0001
C	-3.1879	0.3542	0
C	-1.9838	-0.3411	0.0001
C	-0.7802	0.3538	0.0002
C	0.4234	-0.341	0.0002
C	1.6274	0.3544	0.0002
C	1.6278	1.7442	0.0002
C	2.8312	-0.3405	0.0002
C	2.8308	-1.7308	0.0001
C	1.627	-2.4263	0
C	0.4233	-1.7314	0.0001

C	-1.9835	-1.7315	0
C	-3.1872	-2.4266	-0.0001
C	-4.3911	-1.7311	-0.0001
C	-4.3916	-0.3408	-0.0001
C	-0.7801	-2.4268	0
H	-0.7805	5.5234	0.0002
H	1.2898	4.3288	0.0003
H	-2.8506	4.3286	0.0001
H	-4.0544	2.244	0
H	2.4938	2.2443	0.0003
H	3.6972	0.1594	0.0002
H	3.6969	-2.2307	0
H	1.6272	-3.4263	0
H	-3.1873	-3.4266	-0.0001
H	-5.2577	0.159	-0.0001
H	-0.7801	-3.4268	0

Table S3. Functional and basis groups and Cartesian coordinates in Gaussian optimization tasks for N=4-2TSCs.

#p opt pbe1pbe def2svp em=gd3bj			
0 1			
C	1.1479	-8.1115	0.0002
C	0.3617	-9.4355	0.0002
C	1.117	-10.7781	0.0005
C	2.6577	-10.7973	0.0005
C	3.4424	-9.4726	0.0001
C	2.687	-8.1309	0.0005
C	3.4128	-12.1399	0.0003
C	2.6266	-13.464	0.0007
C	1.086	-13.444	0.0001
C	0.3308	-12.101	0.0003
C	-1.1791	-9.4152	0.0001
C	-1.9676	-10.7386	0.0003
C	-1.2104	-12.081	0.0002
C	-1.9945	-13.4049	-0.0002
C	-3.5339	-13.3882	0
C	-4.295	-12.0494	-0.0002
C	-3.5114	-10.7212	0.0001
C	-1.2397	-14.7477	-0.0004
C	0.3001	-14.7676	0.0001
C	1.0532	-16.1111	-0.0001
C	2.5932	-16.1314	0.0002
C	3.3801	-14.8076	0.0002

C	2.8142	-0.124	0.0001
C	3.5612	-1.4711	0
C	5.1012	-1.4973	0.0003
C	5.8943	-0.1768	0.0006
C	5.147	1.17	0
C	3.6071	1.1964	0.0002
C	7.4344	-0.2033	0.0004
C	8.1809	-1.5505	0.0005
C	7.3881	-2.8709	0.0001
C	5.8482	-2.8443	0.0002
C	2.7689	-2.7926	0.0001
C	3.5157	-4.1402	-0.0003
C	5.0557	-4.1654	0.0002
C	5.8036	-5.5118	0.0002
C	5.013	-6.8328	0.0002
C	3.4739	-6.8081	0
C	2.7244	-5.4625	-0.0002
C	7.3427	-5.5381	-0.0001
C	8.1348	-4.218	0
C	9.6741	-4.2451	0.0003
C	10.4672	-2.9251	0.0002
C	9.7207	-1.5779	0.0002
C	-12.925	-5.5524	0.0003
C	-13.7262	-4.2376	0.0002
C	-15.2655	-4.2756	0
C	-16.0031	-5.6279	0.0002
C	-15.2017	-6.9419	-0.0001
C	-13.6631	-6.9033	-0.0001
C	-17.5418	-5.6659	-0.0002
C	-18.3432	-4.3517	-0.0002
C	-17.6064	-2.9996	0.0004
C	-16.0672	-2.9612	0.0003
C	-12.9889	-2.8854	0.0003
C	-13.791	-1.5704	0.0004
C	-15.3302	-1.6087	0.0001
C	-16.1331	-0.2946	0.0007
C	-15.3968	1.0583	0
C	-13.8573	1.0968	-0.0001
C	-13.0545	-0.2178	0.0004
C	-17.6725	-0.3333	-0.0003
C	-18.409	-1.6857	-0.0002
C	-19.9484	-1.7241	-0.0003

C	-20.6847	-3.0766	-0.0007
C	-19.882	-4.3903	-0.0001
C	-6.6375	-10.7416	0.0003
C	-8.1801	-10.7765	0
C	-8.9216	-12.1263	0.0001
C	-8.1217	-13.4413	0.0001
C	-6.5825	-13.4075	0.0003
C	-5.8378	-12.0596	0.0001
C	-8.8607	-14.7918	0
C	-10.3998	-14.8266	-0.0002
C	-11.2009	-13.5116	-0.0008
C	-10.4618	-12.1614	0.0001
C	-8.9822	-9.4611	0.0004
C	-10.5229	-9.4963	-0.0003
C	-11.2628	-10.8467	0.0006
C	-12.8029	-10.8821	0
C	-13.6033	-9.5667	0.0004
C	-12.8629	-8.2172	0.0005
C	-11.3238	-8.1811	0.0002
C	-13.542	-12.2326	0.0001
C	-12.7405	-13.5476	0.0002
C	-13.4781	-14.8988	-0.0004
C	-12.6768	-16.2138	-0.0003
C	-11.1376	-16.1776	-0.0002
H	0.5908	-7.1168	0.0003
H	4.5825	-9.4846	0.0008
H	4.5528	-12.1533	0.0008
H	-1.7356	-8.4203	0.0008
H	-4.1127	-14.3702	-0.0006
H	-4.0714	-9.728	0.0009
H	-1.8213	-15.728	-0.0001
H	0.4706	-17.091	-0.0007
H	3.1503	-17.1259	0.0001
H	4.5201	-14.8219	0.0009
H	1.6744	-0.1043	-0.0004
H	5.7336	2.1475	0.0012
H	3.054	2.1933	-0.0005
H	8.0216	0.7738	0.0006
H	1.6291	-2.7732	-0.0003
H	5.5668	-7.8294	0.0015
H	1.5845	-5.4453	-0.0007
H	7.8958	-6.5349	-0.0005

H	10.2265	-5.2423	-0.0002
H	11.6071	-2.9451	0.0011
H	10.3081	-0.6011	0.0005
H	-11.7853	-5.526	-0.0006
H	-15.7468	-7.9432	-0.0004
H	-18.0875	-6.6668	0.0008
H	-11.8493	-2.8579	0.0008
H	-15.9915	2.0311	0.0011
H	-13.312	2.0979	0.0007
H	-11.9149	-0.1896	-0.0006
H	-18.2671	0.6394	0.0005
H	-20.5429	-0.7514	-0.0005
H	-21.8244	-3.1051	-0.0007
H	-20.4269	-5.3917	-0.0006
H	-6.0896	-9.7417	0.0001
H	-5.9919	-14.3828	0.0006
H	-8.2684	-15.7661	-0.0002
H	-8.4353	-8.4609	0
H	-14.7433	-9.5917	0.0009
H	-10.7774	-7.1806	-0.0005
H	-14.6817	-12.2588	-0.0004
H	-14.6177	-14.9256	-0.0001
H	-13.2232	-17.2141	-0.0006
H	-10.5441	-17.1511	-0.0016

Table S4. Functional and basis groups and Cartesian coordinates in Gaussian optimization tasks for N=6cTSCs.

#p opt pbe1pbe def2svp em=gd3bj			
0 1			
C	-4.7386	2.4421	0
C	-3.9686	1.1085	0
C	-2.4286	1.1085	0
C	-1.6586	2.4421	0
C	-2.4286	3.7758	0
C	-3.9686	3.7758	0
C	-0.1186	2.4421	0
C	0.6514	1.1085	0
C	-0.1186	-0.2252	0
C	-1.6586	-0.2252	0
C	0.6514	-1.5589	0
C	2.1914	-1.5589	0
C	2.9618	-0.2276	0.046
C	2.1914	1.1085	0

C	-2.4286	-1.5589	0
C	-3.9686	-1.5589	0
C	-4.7386	-0.2252	0
C	-0.1186	-2.8926	0
C	-1.6586	-2.8926	0
C	-4.7386	-2.8926	0
C	-3.9686	-4.2262	0
C	-2.4286	-4.2262	0
H	-5.8786	2.4421	0
H	-1.8586	4.7631	0
H	0.4514	3.4294	0
H	2.7614	-2.5456	-0.0341
H	2.7622	2.0947	-0.034
H	-5.8786	-0.2252	0
H	0.4514	-3.8798	0
H	-5.8786	-2.8926	0
H	-4.5386	-5.2135	0
H	-1.8586	-5.2135	0
C	5.4253	-1.5733	0
C	6.9651	-1.5464	0
C	7.7117	-0.1995	0
C	6.9185	1.1205	0
C	5.3787	1.0937	0
C	4.6321	-0.2533	0
C	7.6651	2.4674	0
C	9.2049	2.4943	0
C	9.998	1.1743	0
C	9.2514	-0.1726	0
C	11.5378	1.2012	0
C	12.2844	2.5481	0
C	11.4936	3.8673	0.046
C	9.9515	3.8412	0
C	10.0446	-1.4927	0
C	9.298	-2.8396	0
C	7.7582	-2.8665	0
C	12.331	-0.1189	0
C	11.5844	-1.4658	0
C	10.0911	-4.1596	0
C	11.6309	-4.1327	0
C	12.3775	-2.7858	0
H	4.8726	-2.5704	0
H	4.7916	2.0708	0

H	7.078	3.4446	0
H	13.4237	2.5683	-0.0341
H	9.3657	4.8186	-0.034
H	7.2055	-3.8635	0
H	13.4708	-0.099	0
H	9.5385	-5.1567	0
H	12.2181	-5.1099	0
H	13.5173	-2.7659	0
C	11.2756	11.9363	0
C	12.0917	10.6303	0
C	13.6307	10.684	0
C	14.3537	12.0438	0
C	13.5376	13.3498	0
C	11.9986	13.296	0
C	15.8928	12.0975	0
C	16.7088	10.7915	0
C	15.9859	9.4318	0
C	14.4468	9.378	0
C	16.8019	8.1258	0
C	18.341	8.1795	0
C	19.0645	9.5369	0.046
C	18.2479	10.8453	0
C	13.7238	8.0183	0
C	12.1848	7.9645	0
C	11.3687	9.2705	0
C	16.079	6.766	0
C	14.5399	6.7123	0
C	11.4618	6.6048	0
C	12.2778	5.2988	0
C	13.8169	5.3525	0
H	10.1363	11.8965	0
H	14.0728	14.3563	0
H	16.428	13.1041	0
H	18.9451	7.2133	-0.0341
H	18.7839	11.8508	-0.034
H	10.2294	9.2307	0
H	16.6831	5.7993	0
H	10.3225	6.565	0
H	14.421	4.3858	0
C	11.8256	16.1771	0
C	11.0556	17.5108	0
C	9.5156	17.5108	0

C	8.7456	16.1771	0
C	9.5156	14.8434	0
C	11.0556	14.8434	0
C	7.2056	16.1771	0
C	6.4356	17.5108	0
C	7.2056	18.8444	0
C	8.7456	18.8444	0
C	6.4356	20.1781	0
C	4.8956	20.1781	0
C	4.1252	18.8468	0.046
C	4.8956	17.5108	0
C	9.5156	20.1781	0
C	11.0556	20.1781	0
C	11.8256	18.8444	0
C	7.2056	21.5118	0
C	8.7456	21.5118	0
C	11.8256	21.5118	0
C	11.0556	22.8455	0
C	9.5156	22.8455	0
H	12.9656	16.1771	0
H	8.9456	13.8561	0
H	6.6356	15.1898	0
H	4.3256	21.1648	-0.0341
H	4.3248	16.5245	-0.034
H	12.9656	18.8444	0
H	6.6356	22.4991	0
H	12.9656	21.5118	0
H	11.6256	23.8327	0
H	8.9456	23.8327	0
C	1.6617	20.1925	0
C	0.122	20.1656	0
C	-0.6247	18.8187	0
C	0.1685	17.4987	0
C	1.7083	17.5256	0
C	2.4549	18.8725	0
C	-0.5781	16.1518	0
C	-2.1179	16.1249	0
C	-2.911	17.4449	0
C	-2.1644	18.7918	0
C	-4.4508	17.4181	0
C	-5.1974	16.0711	0
C	-4.4065	14.7519	0.046

C	-2.8645	14.778	0
C	-2.9576	20.1119	0
C	-2.211	21.4588	0
C	-0.6712	21.4857	0
C	-5.244	18.7381	0
C	-4.4973	20.085	0
C	-3.0041	22.7788	0
C	-4.5439	22.752	0
C	-5.2905	21.405	0
H	2.2144	21.1896	0
H	2.2954	16.5484	0
H	0.009	15.1746	0
H	-6.3367	16.051	-0.0341
H	-2.2786	13.8006	-0.034
H	-0.1185	22.4827	0
H	-6.3838	18.7182	0
H	-2.4514	23.7759	0
H	-5.131	23.7291	0
H	-6.4303	21.3851	0
C	-4.1886	6.683	0
C	-5.0046	7.9889	0
C	-6.5437	7.9352	0
C	-7.2667	6.5755	0
C	-6.4506	5.2695	0
C	-4.9116	5.3232	0
C	-8.8058	6.5217	0
C	-9.6218	7.8277	0
C	-8.8988	9.1875	0
C	-7.3598	9.2412	0
C	-9.7149	10.4934	0
C	-11.254	10.4397	0
C	-11.9775	9.0824	0.046
C	-11.1609	7.774	0
C	-6.6368	10.6009	0
C	-5.0977	10.6547	0
C	-4.2817	9.3487	0
C	-8.9919	11.8532	0
C	-7.4529	11.9069	0
C	-4.3747	12.0144	0
C	-5.1908	13.3204	0
C	-6.7299	13.2667	0
H	-3.0493	6.7227	0

H	-6.9858	4.2629	0
H	-9.341	5.5152	0
H	-11.8581	11.4059	-0.0341
H	-11.6969	6.7684	-0.034
H	-3.1424	9.3885	0
H	-9.596	12.82	0
H	-3.2354	12.0542	0
H	-7.334	14.2334	0
H	20.2018	9.5745	0.114
H	-13.1148	9.0447	0.114

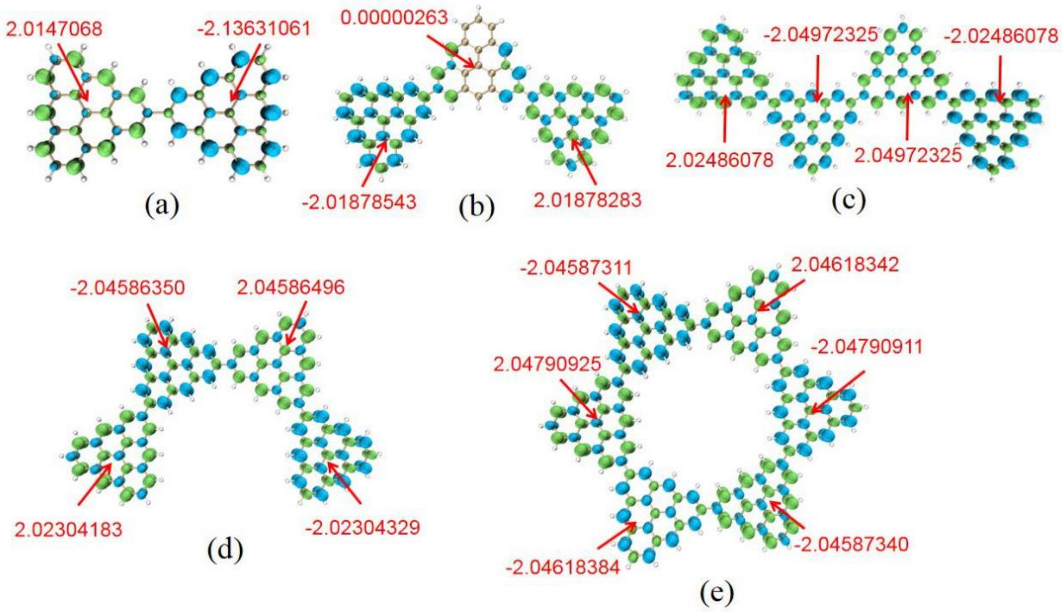

Figure S4. The spin density of the triangulene spin chains and the corresponding spin population of each segment. (Blue isoplanes represent places with negative spin density and green isoplanes represent places with positive spin density).

Thanks to the "magic" shape of the triangle structure, the electrons would "rotate" more easily in one direction, with a net magnetic moment, and thus triangulene was predicted to be magnetic. In order to investigate the magnetic properties of TSCs, it is necessary to calculate the distribution of unpaired electrons on triangulene in three-dimensional space, namely, the spin density, which represents Alpha electron density minus Beta electron density:

$$spindensity = \rho_{e_{Alpha}} - \rho_{e_{Beta}} \quad (1)$$

When the spin density is positive, there are more Alpha electrons than Beta electrons. Negative means there are more Beta electrons than Alpha electrons. FIG. S2a shows that the spin density of N=2TSCs is mainly distributed on the carbon atom at the prominent position of the zigzag edge of triangulene, and the positive and negative isoplanes of the whole molecule are symmetrical. The green isoplane of triangulene on the left is larger than the blue isoplane, and the green isoplane of triangulene on the right is larger than the blue isoplane, and the isoplanes are almost the same size. Almost therefore, the overall spin density of N=2TSCs is 0. In order to investigate the number of single electrons on the fragment, we calculated the spin population of the fragment. The spin population value of the positive spin density dominated on the left side was 2.0147068, indicating that there

were two more Alpha electrons than Beta electrons on the triangulene , which was very close to the two single electrons predicted by the theory. On the contrary, On the right side, the spin population with negative spin density is -2.1363, indicating that there are 2 fewer Alpha electrons than Beta electrons in the triangulene. After adding a triangulene unit, the negative spin density on the leftmost triangulene was dominant, and the spin population value showed that Alpha electrons were 2 less than Beta electrons, and the right side was opposite. Moreover, the spin density near the left side of the middle triangulene was positive, and the spin population was opposite. The spin density near the right side was negative, and the spin density of the middle triangulene was 0. Spin population values show almost no single electron distribution. On the whole, the spin density of N=3TSCs is also 0, as shown in Figure S2b. After adding another triangulene unit, the size of the TSCs further increases, and the molecule can be regarded as the result of the connection of two N=2TSCs. Figure S2c shows that the spin density distribution is similar to that of N=2TSCs, and the spin population value of each triangulene fragment is also very similar. After changing the connection mode of the fourth triangulene unit, based on the previous analysis, it can be speculated that the overall spin density of the triangulene spin chain is 0, and neighboring triangulene units have opposite spin population. This conjecture is confirmed by the spin density and spin population of N=6cTSCs, as shown in Figure S2e. Because N-3TSCs are connected to triangulene with opposite spin population on both sides, there are both positive spin density distributions and negative spin density distributions, and the difference between the two is almost 0. Since the two sides have opposite spin densities, it can be guessed that the carbon atom near the junction point of triangulene has a higher spin density.

To compare the size of spin density on each atom, it is necessary to consider the number of single electrons on each atom. We calculate the contribution of the spin population of the atom to the single electron, which is plotted in the way of atom coloring, as shown in Figure S3. You can clearly see that the carbon atoms with the jagged edges are darker and contribute more to their single electrons. However, such qualitative analysis could not explain the larger contribution of atomic spin population at the triangulene junction point, and the contribution of each atomic electron orbital to single electron should also be investigated from a quantitative perspective. Therefore, we further calculated the atomic spin population of N=2TSCs, as shown in Table S5. Figure S3 is the molecular model of N=2TSCs marked by atomic serial number. The serial number of carbon atoms at the connection point of two triangulene units is 14,12, 24,28. From Table S5, the absolute value of contribution of their p orbitals to single electrons is close to 0.34. The other atoms in the serrated edge prominent positions are 1,5,20,22 and 37,39,42,44, whose absolute value of contribution to single electron is between 0.30 and 0.31. The difference of electron spin population in these two positions leads to the opposite spin density on the left and right side of N=3TSCs intermediate triangulene.

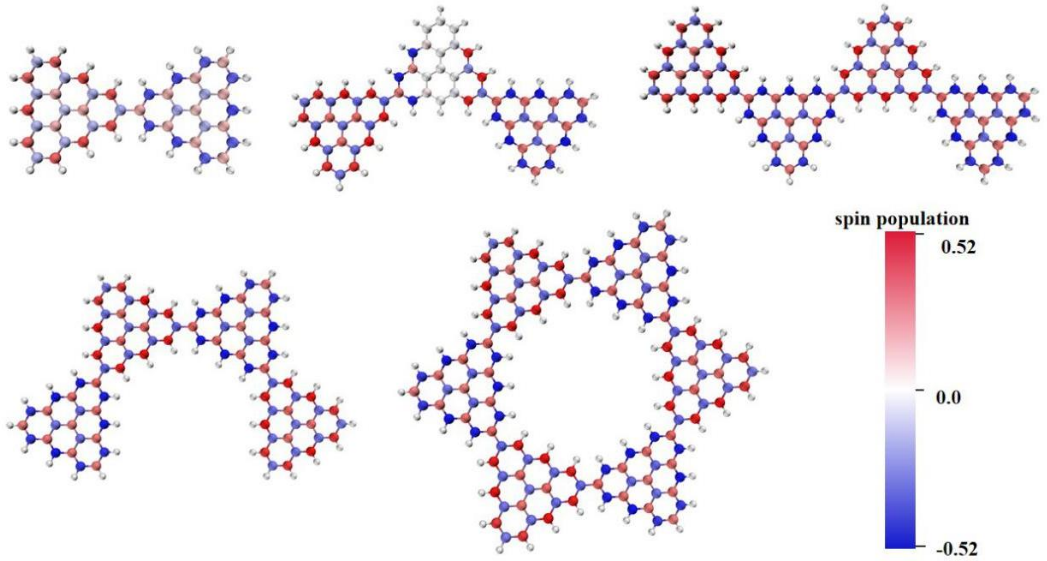

Figure S5. Atomic coloring diagram of the contribution of atomic spin populations to a single electron in a triangulene spin chains, with red representing a positive contribution and blue representing a negative contribution.

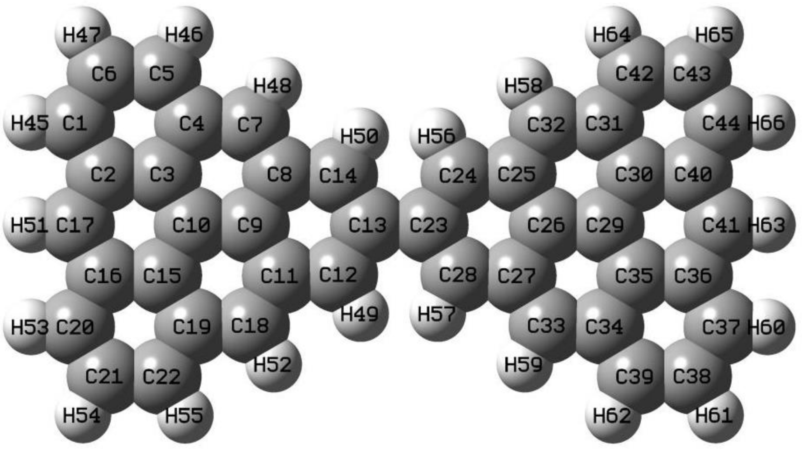

Figure S6. N=2TSCs molecular model, atomic species and corresponding atomic number.

Table S5. Atomic population of N=2TSCs

Atom	electron orbital	Alpha pop.	Beta pop.	Total pop.	Spin pop
	s	1.6418	1.60062	3.24242	0.04118
1(C)	p	1.63438	1.33045	2.96483	0.30394
	d	0.0042	0.00637	0.01057	-0.00216
	s	1.57456	1.60419	3.17875	-0.02963
2(C)	p	1.23748	1.38722	2.6247	-0.14974
	d	0.00685	0.00197	0.00882	0.00488
	s	1.60828	1.58306	3.19134	0.02521
3(C)	p	1.48782	1.32565	2.81347	0.16218
	d	0.0034	0.00533	0.00873	-0.00193
4(C)	s	1.57465	1.60453	3.17918	-0.02988

	p	1.23697	1.38764	2.62461	-0.15067
	d	0.00685	0.00198	0.00883	0.00487
	s	1.64252	1.601	3.24352	0.04152
5(C)	p	1.63624	1.32929	2.96553	0.30695
	d	0.0042	0.00637	0.01057	-0.00217
	s	1.6004	1.62832	3.22873	-0.02792
6(C)	p	1.36924	1.51829	2.88754	-0.14905
	d	0.00729	0.003	0.01029	0.00429
	s	1.66662	1.61282	3.27944	0.0538
7(C)	p	1.6922	1.34405	3.03624	0.34815
	d	0.00469	0.00694	0.01163	-0.00225
	s	1.57369	1.60573	3.17942	-0.03204
8(C)	p	1.22696	1.38546	2.61241	-0.1585
	d	0.00712	0.00199	0.00911	0.00513
	s	1.60971	1.58289	3.1926	0.02681
9(C)	p	1.48905	1.31452	2.80357	0.17453
	d	0.00346	0.00544	0.0089	-0.00199
	s	1.58544	1.60795	3.19338	-0.02251
10(C)	p	1.32579	1.45559	2.78139	-0.1298
	d	0.00604	0.0037	0.00974	0.00234
	s	1.57369	1.60573	3.17942	-0.03204
11(C)	p	1.22696	1.38546	2.61241	-0.1585
	d	0.00712	0.00199	0.00911	0.00513
	s	1.65385	1.60426	3.2581	0.04959
12(C)	p	1.67985	1.34308	3.02293	0.33677
	d	0.00453	0.00681	0.01134	-0.00228
	s	1.57671	1.60871	3.18541	-0.032
13(C)	p	1.25758	1.4296	2.68718	-0.17201
	d	0.00705	0.00187	0.00892	0.00517
	s	1.65385	1.60426	3.2581	0.04959
14(C)	p	1.67985	1.34308	3.02293	0.33677
	d	0.00453	0.00681	0.01134	-0.00228
	s	1.60828	1.58306	3.19134	0.02521
15(C)	p	1.48782	1.32565	2.81347	0.16218
	d	0.0034	0.00533	0.00873	-0.00193
	s	1.57456	1.60419	3.17875	-0.02963
16(C)	p	1.23748	1.38722	2.6247	-0.14974
	d	0.00685	0.00197	0.00882	0.00488
	s	1.66512	1.6115	3.27662	0.05361
17(C)	p	1.69249	1.34275	3.03524	0.34975
	d	0.00465	0.00688	0.01152	-0.00223
18(C)	s	1.66662	1.61282	3.27944	0.0538

	p	1.6922	1.34405	3.03624	0.34815
	d	0.00469	0.00694	0.01163	-0.00225
	s	1.57465	1.60453	3.17918	-0.02988
19(C)	p	1.23697	1.38764	2.6246	-0.15067
	d	0.00685	0.00198	0.00883	0.00487
	s	1.6418	1.60062	3.24242	0.04118
20(C)	p	1.63439	1.33045	2.96483	0.30394
	d	0.0042	0.00637	0.01057	-0.00216
	s	1.6004	1.62832	3.22873	-0.02792
21(C)	p	1.36924	1.51829	2.88754	-0.14905
	d	0.00729	0.003	0.01029	0.00429
	s	1.64252	1.601	3.24352	0.04152
22(C)	p	1.63624	1.32929	2.96553	0.30695
	d	0.0042	0.00637	0.01057	-0.00217
	s	1.6087	1.5767	3.18541	0.032
23(C)	p	1.4296	1.25759	2.68718	0.17201
	d	0.00187	0.00705	0.00892	-0.00517
	s	1.60425	1.65385	3.2581	-0.04959
24(C)	p	1.34308	1.67985	3.02293	-0.33677
	d	0.00681	0.00453	0.01134	0.00228
	s	1.60573	1.57369	3.17942	0.03204
25(C)	p	1.38546	1.22696	2.61242	0.1585
	d	0.00199	0.00712	0.00911	-0.00513
	s	1.58289	1.60971	3.1926	-0.02681
26(C)	p	1.31452	1.48905	2.80357	-0.17453
	d	0.00544	0.00346	0.0089	0.00199
	s	1.60573	1.57369	3.17942	0.03204
27(C)	p	1.38546	1.22696	2.61242	0.1585
	d	0.00199	0.00712	0.00911	-0.00513
	s	1.60425	1.65385	3.2581	-0.04959
28(C)	p	1.34308	1.67985	3.02293	-0.33677
	d	0.00681	0.00453	0.01134	0.00228
	s	1.60795	1.58544	3.19338	0.02251
29(C)	p	1.45559	1.32579	2.78139	0.1298
	d	0.0037	0.00604	0.00974	-0.00234
	s	1.58306	1.60828	3.19134	-0.02521
30(C)	p	1.32565	1.48782	2.81347	-0.16218
	d	0.00533	0.0034	0.00873	0.00193
	s	1.60453	1.57465	3.17918	0.02988
31(C)	p	1.38764	1.23697	2.6246	0.15067
	d	0.00198	0.00685	0.00883	-0.00487
32(C)	s	1.61282	1.66662	3.27944	-0.0538

	p	1.34405	1.6922	3.03624	-0.34815
	d	0.00694	0.00469	0.01163	0.00225
	s	1.61282	1.66662	3.27944	-0.0538
33(C)	p	1.34405	1.6922	3.03624	-0.34815
	d	0.00694	0.00469	0.01163	0.00225
	s	1.60453	1.57465	3.17918	0.02988
34(C)	p	1.38764	1.23697	2.6246	0.15067
	d	0.00198	0.00685	0.00883	-0.00487
	s	1.58306	1.60828	3.19134	-0.02521
35(C)	p	1.32565	1.48782	2.81347	-0.16218
	d	0.00533	0.0034	0.00873	0.00193
	s	1.60419	1.57456	3.17875	0.02963
36(C)	p	1.38722	1.23748	2.6247	0.14974
	d	0.00197	0.00685	0.00882	-0.00488
	s	1.60062	1.6418	3.24242	-0.04118
37(C)	p	1.33045	1.63438	2.96483	-0.30394
	d	0.00637	0.0042	0.01057	0.00216
	s	1.62832	1.6004	3.22873	0.02792
38(C)	p	1.51829	1.36924	2.88754	0.14905
	d	0.003	0.00729	0.01029	-0.00429
	s	1.601	1.64252	3.24352	-0.04152
39(C)	p	1.32929	1.63624	2.96553	-0.30695
	d	0.00637	0.0042	0.01057	0.00217
	s	1.60419	1.57456	3.17875	0.02963
40(C)	p	1.38722	1.23748	2.6247	0.14974
	d	0.00197	0.00685	0.00882	-0.00488
	s	1.6115	1.66512	3.27662	-0.05361
41(C)	p	1.34275	1.69249	3.03524	-0.34975
	d	0.00688	0.00465	0.01152	0.00223
	s	1.601	1.64252	3.24352	-0.04152
42(C)	p	1.32929	1.63624	2.96553	-0.30695
	d	0.00637	0.0042	0.01057	0.00217
	s	1.62832	1.6004	3.22873	0.02792
43(C)	p	1.51829	1.36924	2.88754	0.14905
	d	0.003	0.00729	0.01029	-0.00429
	s	1.60062	1.6418	3.24242	-0.04118
44(C)	p	1.33045	1.63438	2.96483	-0.30394
	d	0.00637	0.0042	0.01057	0.00216
45(H)	s	0.42676	0.44257	0.86933	-0.01581
46(H)	s	0.42681	0.44279	0.86961	-0.01598
47(H)	s	0.43742	0.43118	0.8686	0.00624
48(H)	s	0.42746	0.44464	0.87209	-0.01718

49(H)	s	0.42368	0.44097	0.86465	-0.01729
50(H)	s	0.42368	0.44097	0.86465	-0.01729
51(H)	s	0.42674	0.44409	0.87083	-0.01734
52(H)	s	0.42746	0.44464	0.87209	-0.01718
53(H)	s	0.42676	0.44257	0.86933	-0.01581
54(H)	s	0.43742	0.43118	0.8686	0.00624
55(H)	s	0.42681	0.44279	0.86961	-0.01598
56(H)	s	0.44097	0.42368	0.86465	0.01729
57(H)	s	0.44097	0.42368	0.86465	0.01729
58(H)	s	0.44464	0.42746	0.87209	0.01718
59(H)	s	0.44464	0.42746	0.87209	0.01718
60(H)	s	0.44257	0.42676	0.86933	0.01581
61(H)	s	0.43118	0.43742	0.8686	-0.00624
62(H)	s	0.44279	0.42681	0.86961	0.01598
63(H)	s	0.44409	0.42674	0.87083	0.01734
64(H)	s	0.44279	0.42681	0.86961	0.01598
65(H)	s	0.43118	0.43742	0.8686	-0.00624
66(H)	s	0.44257	0.42676	0.86933	0.01581
	s	80.3692	80.3692	160.7384	0
Total	p	62.41192	62.41192	124.8238	0
	d	0.21888	0.21888	0.43776	0

List of abbreviations

one-photon absorption (OPA)

two-photon absorption (TPA)

triangulene spin chains (TSCs)

electronic circular dichroism (ECD)

carbon nanowires (NWs)

carbon nanotubes (NTs)

cyclic triangulene spin chains (cTSCs)

sum-of-states (SOS)

transition density matrix (TDM)

Time-Dependent Density Functional Theory (TDDFT)

Electrostaticsurface potential (ESP)

transition electric dipole moment (TEDM)

transition magnetic dipole moment (TMDM)

density functional theory (DFT)

Disclaimer/Publisher's Note: The statements, opinions and data contained in all publications are solely those of the individual author(s) and contributor(s) and not of MDPI and/or the editor(s). MDPI and/or the editor(s) disclaim responsibility for any injury to people or property resulting from any ideas, methods, instructions or products referred to in the content.
